# Postmortem Metabolomics Reveal Acylcarnitines as Potential Biomarkers for Fatal Oxycodone-Related Intoxication

**DOI:** 10.3390/metabo12020109

**Published:** 2022-01-25

**Authors:** Albert Elmsjö, Carl Söderberg, Gerd Jakobsson, Henrik Green, Robert Kronstrand

**Affiliations:** 1 Department of Forensic Genetics and Forensic Toxicology, National Board of Forensic Medicine, 587 58 Linköping, Sweden; carl.soderberg@rmv.se (C.S.); Gerd.Jakobsson@rmv.se (G.J.); henrik.green@liu.se (H.G.); robert.kronstrand@rmv.se (R.K.); 2 Department of Biomedical and Clinical Sciences, Division of Clinical Chemistry and Pharmacology, Linköping University, 581 83 Linköping, Sweden

**Keywords:** metabolomics, biomarkers, postmortem, acylcarnitine, death investigation, forensic sciences, β-oxidation, oxycodone, opioids

## Abstract

Postmortem metabolomics has recently been suggested as a potential tool for discovering new biological markers able to assist in death investigations. Interpretation of oxycodone concentrations in postmortem cases is complicated, as oxycodone tolerance leads to overlapping concentrations for oxycodone intoxications versus non-intoxications. The primary aim of this study was to use postmortem metabolomics to identify potential endogenous biomarkers that discriminate between oxycodone-related intoxications and non-intoxications. Ultra-high performance liquid chromatography-quadrupole time-of-flight mass spectrometry data from 934 postmortem femoral blood samples, including oxycodone intoxications and controls positive and negative for oxycodone, were used in this study. Data were processed and evaluated with XCMS and SIMCA. A clear trend in group separation was observed between intoxications and controls, with a model sensitivity and specificity of 80% and 76%. Approximately halved levels of short-, medium-, and long-chain acylcarnitines were observed for oxycodone intoxications in comparison with controls (*p* < 0.001). These biochemical changes seem to relate to the toxicological effects of oxycodone and potentially acylcarnitines constituting a biologically relevant biomarker for opioid poisonings. More studies are needed in order to elucidate the potential of acylcarnitines as biomarker for oxycodone toxicity and their relation to CNS-depressant effects.

## 1. Introduction

Metabolomics strives at quantifying as many low weight molecules as possible within a well determined biological sample, i.e., the metabolome [1]. Metabolomics has proven suitable both for biomarker discovery and for generating new biological hypotheses, and since its introduction has been applied within several of different scientific fields [2]. So far, the number of metabolomics applications used for death investigations are limited, and most efforts have focused on using metabolomics for predicting time since death [3]. However, we recently demonstrated on autopsy cases with pneumonia that postmortem metabolomics could potentially aid in determining the cause of death [4]. Postmortem metabolomics offers a new and unique possibility within the field of forensic toxicology as the postmortem metabolome probably reflects the events leading up to death, the events after death, and potentially also the cause of death.

Overconsumption of opioids can potentially produce life-threatening respiratory depression through their action on μ-opioid receptors. Studies indicates that opioids decrease the human response to hypoxia, which might lead to irregular breathing and, for some cases, complete cessation of rhythmic respiratory function [5]. Other CNS-depressants such as benzodiazepines and ethanol can lead to harmful synergistic effects, further increasing the risk of respiratory depression [6,7]. Oxycodone is a semi-synthetic opioid that is commonly prescribed due to its analgesic properties. Between 2001 and 2015, there was a significant increase in prescription opioid overdose deaths [8,9]. Compared to other Nordic countries, Sweden has experienced a consistent increase in the prevalence of prescribed oxycodone [10]. Expanding the use of oxycodone has led to a higher count of unintentional overdose deaths, and simultaneous use of alcohol and other CNS-depressants may further add to this risk. However, interpretation of oxycodone concentrations in postmortem cases is complicated, as oxycodone tolerance leads to overlapping concentrations for oxycodone intoxications and non-intoxications [11,12].

Hence, the aim of this study was to investigate if postmortem metabolomics could be used to identify metabolic differences between intoxication cases positive for oxycodone in comparison to two control groups, positive and negative for oxycodone. Furthermore, this study aimed to link potential identified biomarkers to hypoxia and the toxicological effect of oxycodone. We found a significant decrease in femoral blood for 25 acylcarnitines in intoxication cases in comparison to control cases. The homeostatic imbalance of acylcarnitines could potentially be linked to respiratory depression and the effect of oxycodone intoxication.

## 2. Results

Postmortem metabolomics provides a biochemical overview of the postmortem metabolome. This overview might provide vital insight into the agonal period and the cause of death. In this study, the postmortem metabolome of oxycodone positive intoxication cases were compared with two control groups, positive and negative for oxycodone. The primary aims were to find potential biomarkers for oxycodone intoxications and to investigate if these biomarkers could be linked to respiratory function and hypoxia.

### 2.1. Study Cohort

A demographic overview of the 934 autopsy cases included in this study are presented in Table 1. A skewed demography was observed between oxycodone intoxications and controls (Table 1). In general, the oxycodone-related intoxication cases included more females, were younger, and had a higher body mass in comparison to the two control groups. In addition, other CNS-depressants, such as benzodiazepines, were more common for oxycodone-related intoxications in comparison to the two control groups. In Supplementary Table S1, all analytical findings of other opioids and common prescribed CNS-depressants are presented.

### 2.2. Multivariate Models

Out of 934 autopsy cases, 625 were used in a training set for model building. A total number of 1397 chromatographic peaks with specific accurate masses and retention times were included in the PCA model, as seen in Supplementary Figure S1. The PCA model showed no clear outliers or unwanted trends related to gender, age, or BMI. Furthermore, no trend related to acquisition date could be observed, indicating no or limited batch-to-batch variation. In addition to this, the internal standards in the blank whole blood samples analyzed over the whole study period (*n* = 4065) showed an absolute area variation of <26% (CV), a maximum mass accuracy deviation of 5.7 ppm, and a standard deviation for the retention times less than 2.5 s, altogether indicating adequate data quality. 

**Figure 1:**
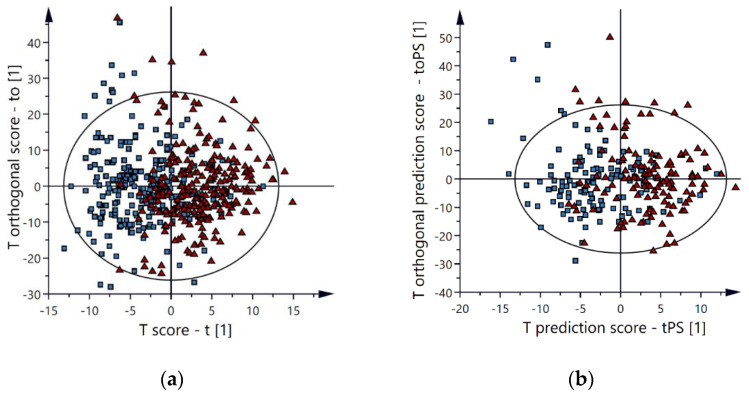
OPLS-DA score plots. An apparent group separation is observed between oxycodone intoxications (▲) and the positive controls (■) for both the training set (**a**) and the validation set (**b**) (R2 = 0.41, Q2 = 0.21).

All chromatographic peaks in the PCA model except chromatographic peaks belonging to oxycodone, noroxycodone, oxycodol, and noroxycodol (including different forms of adducts, isotopes and insource fragments) were then used in an OPLS-DA model in order to identify potential markers able to differentiate oxycodone-related intoxication from oxycodone nonrelated intoxication. In the final OPLS-DA model, without any oxycodone-related peaks, an apparent group separation was observed between oxycodone intoxications and positive control cases, as seen in Figure 1a. The OPLS-DA model described the data well, and the cross-validation score was adequate, indicating that the model was reproducible (R2 = 0.41, Q2 = 0.21).

The remaining 309 autopsy cases were used in a validation set enabling a full validation of the multivariate model, thereby minimizing the risk of overfitting models. The group separation for the validation set was comparable to the training set, as seen in Figure 1b. For the validation set, the model’s sensitivity and specificity were 80% and 74%, while the sensitivity and specificity were 80% and 80% for the training set. In depth analysis of false positives (positive controls classified as intoxications by the multivariate model) showed that several autopsy cases had high levels of oxycodone (or other opioids), and/or ethanol or benzodiazepines even though the primary cause of death was not intoxication (e.g., hanging or drowning). In depth analysis of false negatives (oxycodone-related intoxications classified as positive controls by the multivariate model) showed that several autopsy cases had low levels of oxycodone but high levels of other non-CNS-depressant drugs such as acetaminophen, as seen in Supplementary Table S2. 

### 2.3. Metabolites of Importance

**Figure 2:**
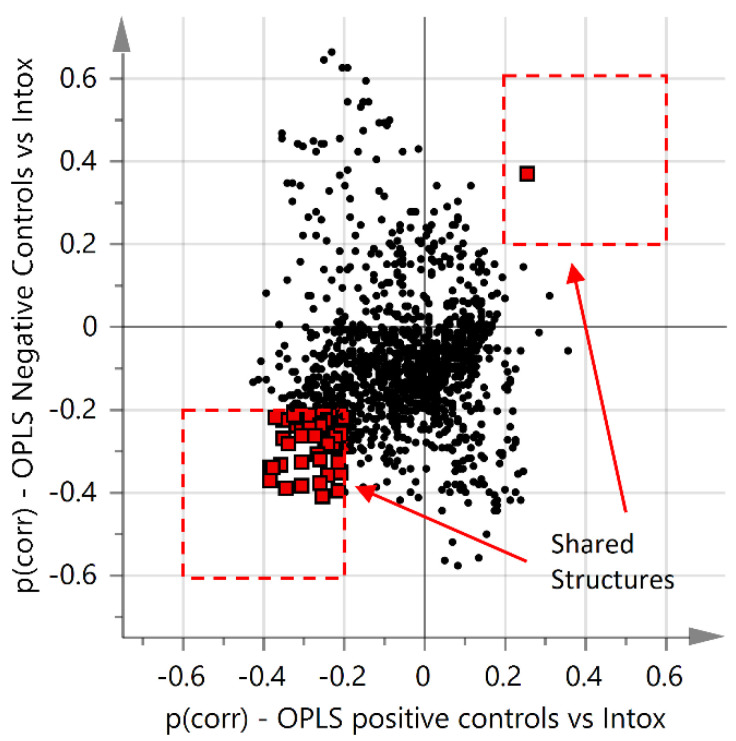
Shared and unique structure plot (SUS-plot). Shared metabolites between the two OPLS-DA models of intoxication vs. positive controls and intoxication vs. negative controls with a *p*(corr) > 2 or *p*(corr) < −2 are highlighted as red squares.

Due to the skewed demographics, several supervised OPLS-models were built in order to ensure interpretation of the results was not affected by the systematic differences. Within each separate group, no or limited trends were observed for the supervised OPLS-models based on gender and BMI (Q2 < 0.028, data not shown). However, several chromatographic peaks correlated with age, and an OPLS model based on age as a y-variable for the negative control is presented in the supplementary material, Supplementary Figure S2. In addition, to further evaluate the influence of age, a new age matched OPLS-DA model was developed. In this model, two thirds of the samples with an age <56 years in the intoxication and two thirds of the samples with an age >56 years in the positive controls were excluded. In both groups, a mean of 56 years was acquired and an age mean comparison with student’s *t*-test gave a *p*-value of 0.92. The age matched OPLS-DA score plot showed similar results to the model using the entire training set but with a reduced predictive power. The corresponding volcano plot of variables of importance over *p*(corr) showed that acylcarnitines were the most prominent metabolites. The OPLS-DA score plot and volcano plot are presented in the supplementary material, as seen in Figure S3. Even so, in order not to neglect the skewed demographics, only features important for discriminating intoxications from both control groups were considered, and a criteria of *p*(corr) > 0.2, or *p*(corr) < −0.2 was used. This is illustrated in a shared and unique structure plot (SUS-plot), as seen in Figure 2. Only chromatographic peaks significantly different between intoxication and both control groups were considered, which are highlighted as red squares in Figure 2.

Furthermore, to ensure that postmortem interval (PMI) differences were not neglected, an age and PMI compensated model was developed, as seen in Figure S4. An increased group separation was observed between intoxication cases versus positive controls but with a reduced model robustness. In addition, a postmortem degradation modified OPLS-model was developed. In this model, all autopsy cases where the forensic pathologist had documented a beginning decomposition, (e.g., some/medium/extensive decomposition or maggots), were excluded. A similar trend in group separation was observed, and no apparent differences in those chromatographic peaks that were important for group discrimination were observed, as seen in Supplementary Figure S5. 

**Figure 3:**
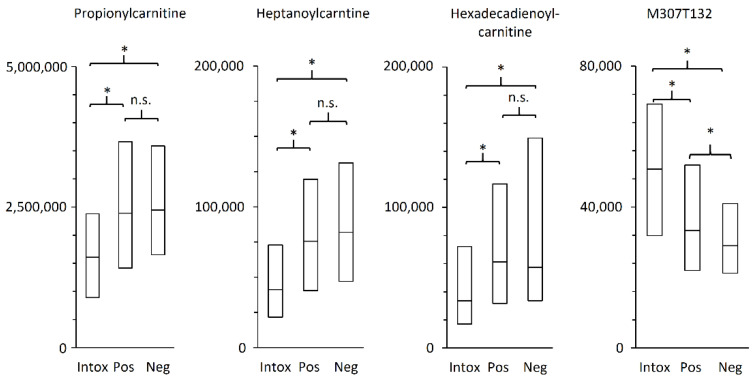
Boxplots of normalized area intensities with first, second, and third quartile. The three identified metabolites illustrates that short-, medium-, and long-chain acylcarnitines are affected in a similar manner. * *p*-value < 0.001 and n.s. non-significant.

All chromatographic peaks identified and important for group separation belonged to the biochemical class acylcarnitines, as seen in Table 2. All acylcarnitines were significantly decreased for intoxication cases compared to control cases. For example, propionylcarnitine, heptanoylcarnitine, and hexadecadienoylcarnitine that belong to short-, medium-, and long-chain acylcarnitines, respectively, all had a significant difference in fold change of approximately ½ for intoxication in comparison to positive controls, as seen in Figure 3. One unidentified chromatographic peak with *m*/*z* 307.122 was significantly higher in the intoxication group in comparison with the controls.

## 3. Discussion

In simple terms, postmortem metabolomics gives a vast overview of the biochemical composition in a given metabolome after death. That overview has primarily been used for postmortem interval (PMI) determination [3]. However, postmortem metabolomics might also provide vital information about the agonal period as well as the biological processes leading up to death [4]. Interpretation of oxycodone concentrations in postmortem cases is complicated due to tolerance and overlapping concentrations for fatal and non-fatal levels. Therefore, this study aimed at identifying biomarkers able to discriminate between intoxication and non-intoxications and possibly link these changes to respiratory depression and hypoxia. 

### 3.1. Study Strengths and Limitations 

Due to the sheer number of autopsy cases, this study was able to use a two-sets study design, with a training set that was cross-validated and an untouched validation set enabling an external validation. Using a two-sets study design to fully validate the multivariate model gave a strong foundation for this postmortem metabolomics investigation. 

Postmortem samples are bound to show high inter-individual variations, as death itself and time since death most likely result in extensive biochemical changes that might be unrelated to the cause of death [14]. The PMI was only known for a small portion of the included autopsy cases, making it difficult to interpret PMI’s impact on the multivariate models. However, as suggested by Chigine et al., a PMI control group was created, as seen in Figure S4 [14]. In this age and PMI-compensated model, an improved group separation was observed. Unfortunately, the model robustness decreased, probably owing to the reduced number of samples. In addition, the multivariate model only including samples without decomposition showed similar results to the previous models. The discriminative power of the OPLS models with intoxications versus controls were highly intriguing, and similar results were observed both for the training set and the validation set, confirming that no model overfitting occurred. In addition, as neither acquisition date nor PMI or postmortem degradation seemed to skew or conceal any results, the study data were considered robust and reliable. 

It is important to mention that all supervised OPLS models assume that the group classification is perfect. For this study, perfect diagnostic accuracy by the forensic pathologist is assumed. We have not found any studies that estimate the error rate of forensic pathological intoxication diagnosis with regard to the correct classification of involved substances or with regard to other potential differential diagnoses. However, in a clinical setting, it has been estimated that approximately 10–15% of diagnoses are incorrect [15]. While these percentages might not be comparable to a forensic pathological setting, it can also not be assumed that the forensic pathologist is correct by default. Therefore, it is very difficult to evaluate the model’s sensitivity (number of false negative) and the specificity (number of false positives). In addition, as the model is based on biochemical changes, some misclassifications by the model could be due to the same biochemical processes being present but to a lesser extent. In order to evaluate this aspect, a small set of false negatives and false positives were inspected in depth. Several of the positive controls, falsely classified as intoxications, had intoxication as a secondary contributing cause of death. For these cases, a high concentration of oxycodone or other opioids or the presence of other CNS depressants were observed, even though the primary cause of death was something else (e.g., hanging or drowning). Looking at the false negatives, some were intoxications with non-opioid substances (for example acetaminophen). In this aspect, it could thereby be argued that these are not true false positives or false negatives as the metabolic pattern was explainable. 

It is also important to mention that the model includes all intoxication where oxycodone is present, even though the concentration might be rather low and the contribution to the primary cause of death could be questionable. The diagnostic accuracy might have been improved by manually evaluating the intoxication cases and only include cases where the contribution from oxycodone is certain. This approach, however, would have been very tedious, and the final multivariate model would only be able to handle a very small and homogeneous set of samples. In addition, a stringent selection might miss vital results, as it is well known that a combination of oxycodone together with other CNS-depressants further increases the risk of respiratory depression [6,7]. However, it is important in future studies to include more CNS-depressants in order to investigate their general effect on acylcarnitine homeostasis. In any case, some error is to be expected as neither a model nor a forensic pathologist can be expected to have complete diagnostic accuracy. With the current sensitivity and specificity, the model is able to highlight cases that merit a closer review in order to confirm or exclude an intoxication diagnosis.

### 3.2. Acylcarnitines Potential as Biomarker for Oxycodone-Related Intoxications

Acylcarnitines have been linked to a number of age-related diseases where most commonly elevated levels of acylcarnitines are observed. For example, elevated levels of acylcarnitines have been suggested as a marker for cardiovascular disease [16,17] and diabetes [18,19]. However, Jarrell et al. recently demonstrated that long-chain and very long-chain acylcarnitines increase with age [20]. These results are troubling as there is an age-related systematic difference between the oxycodone-related intoxications and positive controls in this study. In order to ensure the validity of the results in this postmortem metabolomics investigation, several multivariate models were developed in order to investigate a possible age bias. In the age-matched OPLS-DA model, an apparent group separation was still observed, and acylcarnitines showed high model impact and correlation, as seen in Supplementary Figure S3. In addition, the age-based OPLS model showed that acylcarnitines had a poor model impact and a low model correlation in comparison to the OPLS-DA models of intoxications versus positive controls, as seen in Figure S4. To bypass the age-related differences, only results differentiating oxycodone intoxications from both control groups are presented. This minimized the confounding effects of age, as there were no age-related differences between intoxications and negative controls. 

Acylcarnitines are considered important metabolic intermediates in mitochondrial metabolism [21], and the decreased levels of acylcarnitines observed in our study could therefore be an effect of oxygen depletion due to the presumed respiratory depression for intoxication cases. The are several relevant in vivo studies that report that hypoxia and ischemia affect the homeostasis of acylcarnitines, thereby supporting our results [22,23]. Bjorndalen et al. uses an animal model with a TTP diet (known inhibitor of β-oxidation and mitochondrial function) with the primary aim to evaluate mitochondrial function and respiratory activity. Interestingly, Bjorndalen et al. observes a hepatic lipid accumulation together with a reduction of short chain acylcarnitines in plasma for the rats on a TTP diet [22]. Similarly, Hal et al. observed drastic decreases in levels of acylcarnitines by measuring total carnitine during neonatal hypoxia [23]. Bruder and Raff also observe similar results in neonatal rats (Postnatal Day 2) during acute hypoxia; however, increased levels of plasma acylcarnitines were observed in older rats (Postnatal Day 60) [24]. Whitmer et al. found decreased concentrations of acylcarnitines together with an accumulation of long-chain acylcarnitines in ischemic and hypoxic rat hearts [25]. Friolet et al. demonstrated increased concentrations of short-chain acylcarnitines in plasma after exhaustive exercise under both hypoxic and normoxic conditions [26]. The observed decreases of acylcarnitines in our study might reflect a prolonged hypoxic state and an increase in oxidative stress, resulting in a depletion of acylcarnitine reserves.

Furthermore, several in vitro studies regarding ischemia in cerebral rat cells show that acylcarnitines (primarily acetylcarnitine) have a neuroprotective effect. Pretreatment with acylcarnitines before induced ischemia showed increased cell survival, increased cytochrome c oxidase activity, and reduced oxidative stress compared to controls, among other protective parameters. Even though the mechanism is not fully understood, there are suggested theories that include maintenance of mitochondrial proteins, protection against oxidative stress, and inhibition of apoptosis [27,28,29,30,31]. As high levels of acylcarnitines are protective in ischemia, perhaps the low level observed in this study with a terminal outcome reflects a sustained ischemia in which the acylcarnitines are consumed. 

Even though controversy exists regarding which acylcarnitines are increased, decreased, or unaltered in whole blood or plasma during hypoxia and ischemia, the potential of postmortem metabolomics for biomarker discovery is unquestionable. Further studies are needed to fully elucidate the potential of acylcarnitines as biomarkers for hypoxia and oxycodone toxicity and acylcarnitines’ relation to age and other CNS-depressants. 

## 4. Materials and Methods

All autopsy cases admitted to the Swedish National Board of Forensic Medicine between late June 2017 until end of October 2020 with femoral blood and with a toxicological screening using high-resolution mass-spectrometry were considered for this study (*n* = 17,008). All intoxications with ICD9-codes 965, 967, 969, 970, 977, 980, and 995 and positive for oxycodone were included in the intoxication group (oxycodone intoxication, *n* = 375). All other autopsy cases positive for oxycodone were grouped into the oxycodone-positive control group (Positive Controls, *n* = 364). A third, oxycodone-negative control group with the inclusion criteria ICD9-codes 994, 958, 933 869, 861, 852, and 804; BMI 18.5–30.0; age 20–75; and the exclusion criteria of intoxications (including insulin, cyanide, and carbon monoxide), diseases potentially affecting the metabolic fingerprint (such as cancer, cardiovascular diseases, sepsis and pneumonia), and signs of alcohol damage (Negative Controls, *n* = 195). Each parameter in the demographic overview was compared between the three groups with the statistical methods χ^2^ and one-way ANOVA.

All autopsy cases were analyzed with a standardized procedure described elsewhere [32]. In short, each femoral blood sample was prepared by protein precipitation (MeCN:EtOH, 90:10), including an addition of three internal standards (amphetamine-d8, diazepam-d5 and mianserin-d3). All samples were injected on a UHPLC-ESI-QToF system (Agilent 6540 QTOF with a Jet Stream interface and an Agilent 1290 Infinity LC instrument). Separation was performed on a C18 column (Waters Acquity, HSS T3 column; 150 mm × 2.1 mm, 1.8 μm) using gradient elution. MS data were collected in positive mode, and the total acquisition time for each sample was 12 min. Each analytical run included a blank drug-free bovine whole blood sample (purchased from a local slaughterhouse) also containing the three internal standards, analyzed in the beginning and at the end of each run.

The raw LC/MS data from the selected autopsy cases were exported to mzData-files using Masshunter. The exported files were loaded into R (2.14) and the XCMS package for peak detection and retention time alignment [33]. In XCMS, the centWave algorithm was used for peak detection using the following parameters: Δ*m*/*z* of 15 ppm, minimum peak width of 3 s, maximum peak width of 20 s, and signal-to-noise threshold of 3 with noise variable set to 1500. Retention time correction was performed using the obiwarp function, and for the grouping, an *m*/*z* width of 0.05, base width of 3, and minimum fraction of 0.8 were used. 

All chromatographic peaks before 90 s and after 660 s were excluded, and the remaining features were normalized in Excel using the probabilistic quotient normalization. All autopsy cases (*n* = 934) were ordered after class and acquisition date, and every third case was assigned to a validation set (*n* = 309) used for external validation, while the remaining were used in a training set (*n* = 625) for model building. All variables were scaled with unit variance, log transformed, and subjected to multivariate analysis using SIMCA 15.0.2 (Umetrics, Umeå, Sweden). Principal component analysis (PCA) was used to give an overview of the data, enabling identification of outliers and observation of trends. PLS models for each variable (i.e., age, gender, weight, length, and BMI) were created to investigate systematic differences in the metabolic profiles. Orthogonal partial least-squares discriminant analysis (OPLS-DA) was used to identify features directly related to oxycodone, i.e., isotopes, different adducts, in-source fragments, and oxycodone metabolites. All oxycodone-related features were excluded, and two OPLS-DA models of intoxication (*n* = 249) vs. positive controls (*n* = 244) and intoxication (*n* = 249) vs. negative controls (*n* = 132) were developed. The OPLS-DA model was used to identify features important for group separation with a potential link to oxycodone toxicity. Variable importance for the projection plots (VIP) and shared unique structure plots (SUS) were used to identify potential biomarkers important for discrimination of oxycodone intoxication. Metabolites with a *p*(corr) > 0.2 or <−0.2 were putatively identified and annotated by matching molecular weight (±5 ppm) with the Human Metabolome Database, ref [34] as well as the METLIN database [35]. For visualization, each metabolites’ normalized mean intensity was also statistically evaluated with univariate analysis using student’s *t*-test. All presented *p*-values were Bonferroni-corrected (Microsoft Office Excel 2013). Several OPLS and OPLS-DA models were developed to check and correct for systematic differences related to age, gender, BMI, PMI, grade of decomposition, and acquisition date. For model specifics, the reader is referred to the Figure legends in the supplemental material. 

Experimental reproducibility was assessed by determination of the coefficients of variation (CV) for the isotope-labeled standards in blank whole blood samples and by visual examination of the score plot from the PCA. All multivariate models has been validated using k-fold cross-validation, and the OPLS-DA model of oxycodone intoxications versus positive controls was also evaluated using external validation. For the cross-validation, samples were divided into seven cross-validation groups (k = 7). Each group was, in turn, kept out of model development, and the kept out part was then predicted by the model. After this, the predictions of the kept out part were compared with the actual values. These steps were repeated until all parts (samples) had been kept out. For all included components, SIMCA computes an overall Q2 = 1 − PRESS/SS, where the prediction error sum of squares (PRESS) is the squared differences between observed and predicted values for the Y-data kept out of the model fitting and SS is the sum of squares of Y. For the external validation, left-out autopsy cases were predicted using the corresponding OPLS-DA model. The model predictability was evaluated by determining the model’s sensitivity and specificity. False positives and negatives in the validation set with a predicted score value above 5 or below −5 were investigated in depth in order to elucidate the usability of the multivariate model.

## Figures and Tables

**Table 1:** Demographic overview of study cohort.

	Oxycodone Intoxications	Positive Controls	Negative Controls	Statistics
*n*	375	364	195	
Females/males	160/215	123/241	44/151	*p* < 0.001 ^1^
Age (yrs)	48 (35–60)	65 (53–74)	47 (30–59)	*p* < 0.001 ^2^
Body weight (kg)	86 (72–100)	76 (62–92)	74(65–83)	*p* < 0.001 ^2^
Body Height (cm)	173 (165–181)	173 (165–179)	176 (170–182)	*p* < 0.001 ^2^
Body Mass Index (kg/m^2^)	29 (25–33)	26 (22–30)	24 (22–26)	*p* < 0.001 ^2^

Data are presented as median with quartile range 25–75% in parentheses. ^1^ *p*-value calculated with Chi^2^ (χ^2^)-test, ^2^ *p*-values calculated between the three groups with one-way ANOVA.

**Table 2:** Metabolite information for identified metabolites.

Metabolites ^1^	Chain Length ^2^	Identifier ^3^	Mean *m*/*z* ^4^	Exact *m*/*z* ^5^	Δ ^6^	% ^7^	*p*-Value ^8^
Acetylcarnitine	C2	M204T119	204.123	204.1230	0.0	0.70	4.6 × 10^−4^
Propionylcarnitine	C3	M218T126	218.139	218.1387	−1.4	0.67	3.5 × 10^−12^
Butyrylcarnitine	C4	M232T167_1	232.155	232.1543	−3.0	0.64	2.8 × 10^−7^
(Iso)valerylcarnitine	C5	M246T226	246.170	246.1700	0.0	0.51	1.6 × 10^−19^
Hexanoylcarnitine	C6	M260T289	260.186	260.1856	−1.5	0.69	8.7 × 10^−8^
Heptanoylcarnitine	C7	M274T349	274.201	274.2013	1.1	0.53	2.6 × 10^−15^
Octanoylcarnitine	C8	M288T405	288.217	288.2169	−0.3	0.55	7.4 × 10^−12^
Nonanoylcarnitine	C9	M302T456_2	302.232	302.2326	2.0	0.69	3.6 × 10^−9^
Decenoylcarnitine	C10:1	M314T459	314.232	314.2326	1.9	0.59	8.8 × 10^−3^
Decanoylcarnitine	C10	M316T504	316.248	316.2482	0.6	0.46	7.1 × 10^−8^
Hydroxyhexadecadiencarnitine	C16:2-OH	M412T568	412.304	412.3057	4.1	0.64	2.7 × 10^−4^
Tetradecadiencarnitine	C14:2	M368T572	368.279	368.2795	1.4	0.55	7.1 × 10^−4^
Dodecanoylcarnitine	C12	M344T573	344.279	344.2795	1.5	0.59	6.4 × 10^−6^
Hydroxyhexadecenoylcarnitine	C16:1-OH	M414T588	414.321	414.3214	1.0	0.64	4.0 × 10^−14^
Tetradecenoylcarnitine	C14:1	M370T591	370.295	370.2952	0.5	0.59	1.3 × 10^−7^
Hexadecadienoylcarnitine	C16:2	M396T601	396.311	396.3108	−0.5	0.55	5.8 × 10^−7^
Hydroxyhexadecanoyl carnitine	C16-OH	M416T606	416.337	416.3371	0.2	0.79	3.7 × 10^−8^
Tetradecenoylcarnitine	C14	M372T608	372.311	372.3108	−0.5	0.64	2.9 × 10^−8^
Linolenylcarnitine	C18:3	M422T611	422.327	422.3265	−1.2	0.69	1.9 × 10^−6^
Hydroxyoctadecanoylcarnitine	C18-OH	M442T617	442.353	442.3527	−0.7	0.65	2.3 × 10^−10^
Linoleylcarnitine	C18:2	M424T628	424.343	424.3421	−2.1	0.78	1.9 × 10^−4^
Unidentified	Na	M307T132	307.122	NA	NA	1.59	3.6 × 10^−10^

^1^ Putative identities according to MSI [13], ^2^ number of carbons, saturations, and alcohols on the ester-group on the acylcarnitines, ^3^ specific identifier for each chromatographic peak, including mass and retention time, ^4^ mean measured accurate mass for [H]^+^ over all samples, ^5^ theoretical monoisotopic mass ^6^ ppm difference between measured and theoretical mass, ^7^ % of median of the normalized peak intensities of the intoxication group divided by the positive control group including both training and validation set, and ^8^ Bonferroni-corrected *p*-values for the log transformed normalized intensities in the intoxication group vs. positive controls.

## Data Availability

Data sharing is not applicable to this article.

## Supplementary Materials

The following are available online at https://www.mdpi.com/article/10.3390/metabo12020109/s1, Figure S1: PCA score plot, Figure S2: Age-based OPLS model, Figure S3: Age-matched OPLS-DA model, Figure S4: Age and PMI-matched OPLS-DA model, Figure S5. Modified OPLS-DA model with regard to decomposition, Table S1: Distribution of commonly prescribed CNS-depressants, Table S2: Cause of death and analytical findings for false positives and false negatives (t > 5 or t < −5).
