# Supplementary material for: Postmortem Metabolomics Reveal Acylcarnitines as Potential Biomarkers for Fatal Oxycodone-Related Intoxication
Source: Metabolites. 2022 Jan 25;12(2):109. doi: 10.3390/metabo12020109 (PMC8878426; doi:10.3390/metabo12020109)
Supplement: Supplementary file 1 [file metabolites-12-00109-s001.zip › metabolites-1531723-supplementary.pdf]

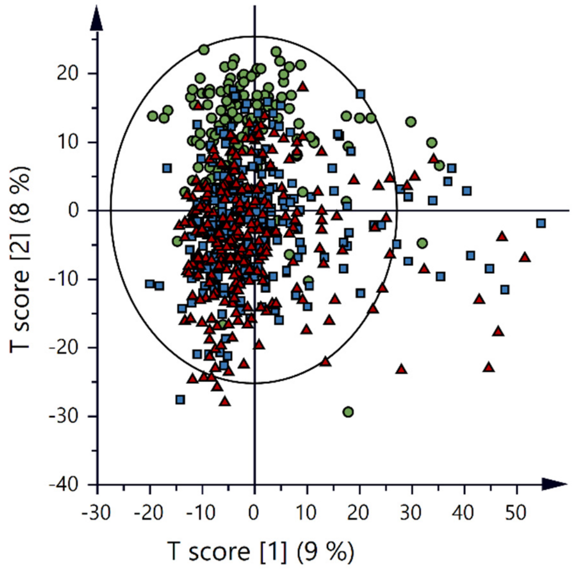


**Figure S1.** ***PCA score plot.*** *Oxycodone intoxications (n=249, ▲), positive controls (n=244, ■) and negative controls (n=132, ●)(R2 = 0.61, Q2 = 0.54).*

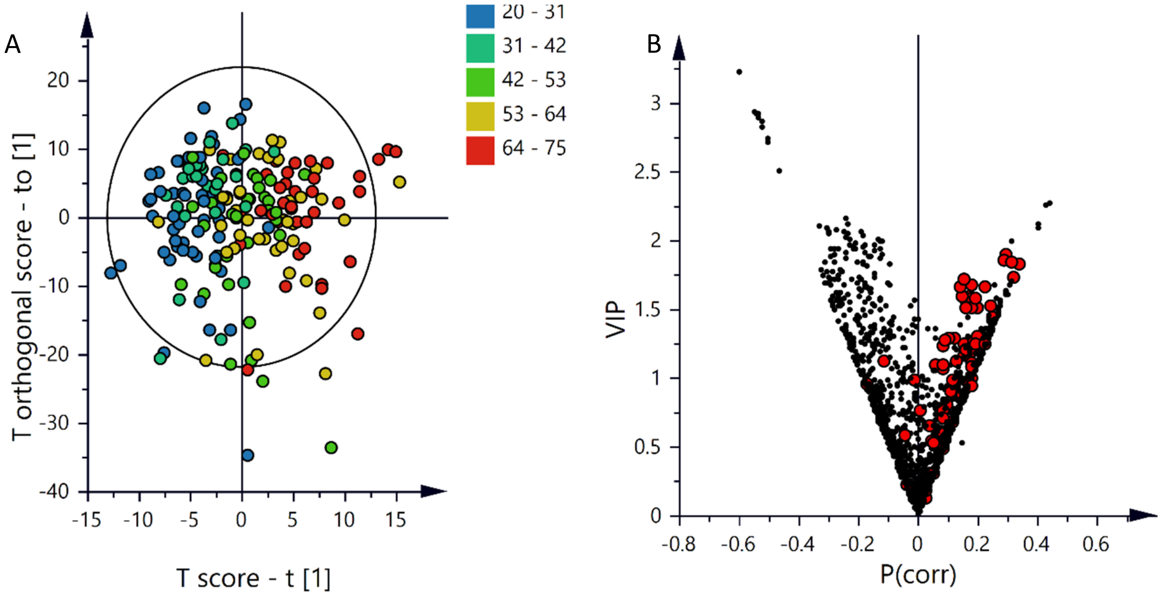


**Figure S2.** ***Age based OPLS model.*** *Figure A is a color coded scoreplot illustrating age related correlations(blue=20-31 years, turquoise=31-42 years , green=42-53 years, yellow=53-64 years, red=64-75 years). The model is based on all autopsy cases from the negative controls (n=195). Figure B is a corresponding volcanoplot, illustrating how acylcarnitines (red circles) impact and correlates with age. Some acylcarntine has a positive corraltion, however the model impact and the correlation is poor (p(corr)<0,2, VIP<1.5), in comparison to acylcarnitines correlation to intoxication. For comparison see Figure 2, Figures S3, S4and S5.*

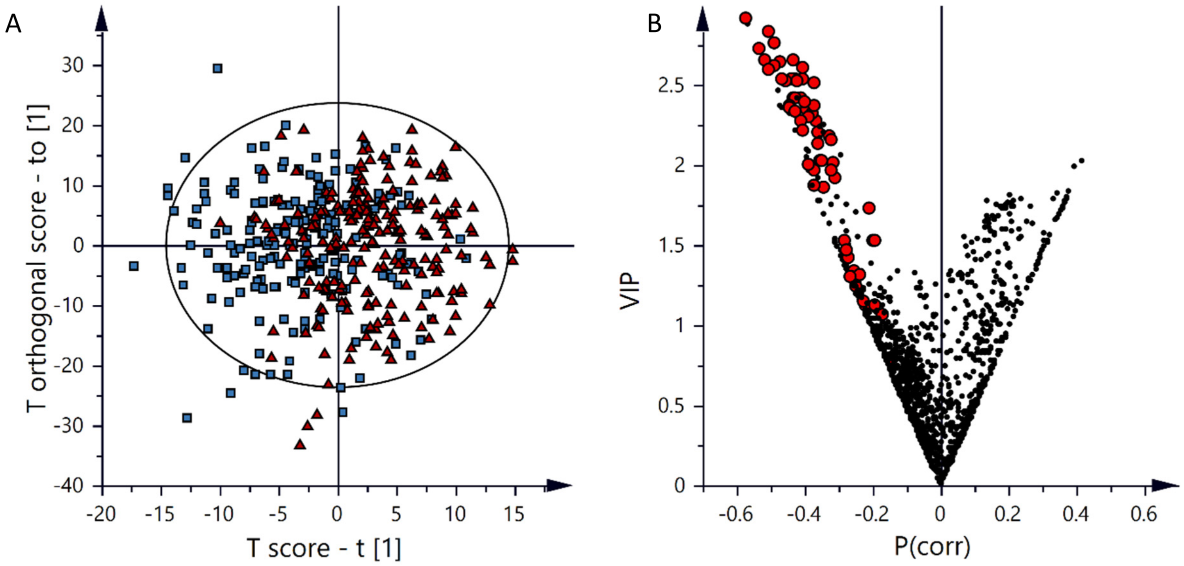


**Figure S3.** ***Age-matched OPLS-DA model.*** *In the age matched OPLS-DA score plot (A), a trend in group separation on a population level is still observed between oxycodone intoxications (n = 207, mean age = 56, ▲) and the positive controls (n = 192, mean age = 56, ■). In addition, a volcanoplot (B) illustrates that acylcarnitines (red circeles) are of high importance for the trend in group separation, where a majority has a p(corr) < -0.2 and VIP > 1,5 (R2 = 0.28, Q2 = 0.12).*

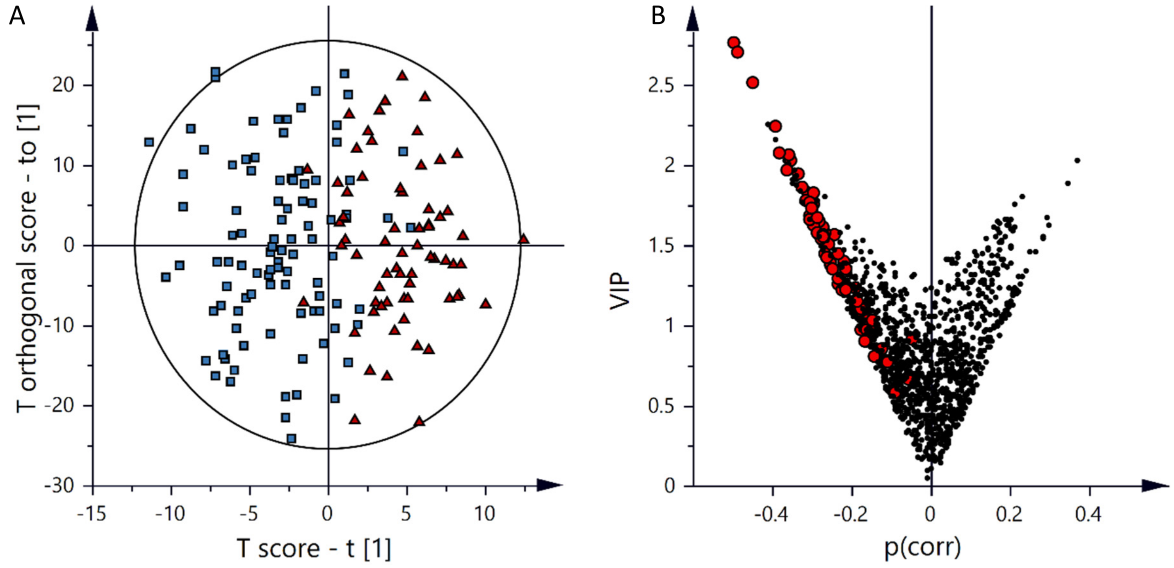


**Figure S4.** ***Age and PMI-matched OPLS-DA model.*** *In the age and PMI matched OPLS-DA score plot (A), a stronger trend in group separation on a population level is observed between oxycodone intoxications (n = 66, ▲) and the positive controls (n =96, ■). In addition, a volcanoplot (B) illustrates that acylcarnitines (red circeles) still are of high importance for the trend in group separation, where a majority has a p(corr) < -0.2 and VIP > 1,5. Mean age and mean PMI were comparable between the groups (p = 0,72 and 0,35 respectively). Sensitivity and specificity for the model were above 90 % however the Q2 were low (R2 = 0.61, Q2 = 0.08).*

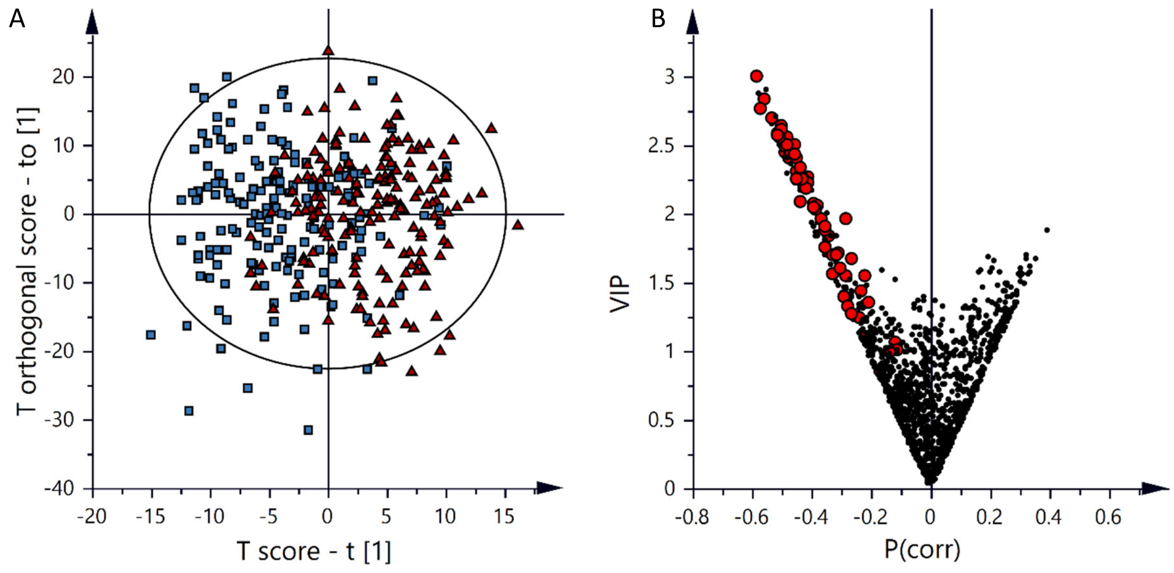


**Figure S5.** ***Modified OPLS-DA model with regard to decomposition.*** *Overview of OPLS-DA model without autopsy cases with tendency of decomposition. In the OPLS-DA score plot (A), a similat trend in group separation on a population level is observed between oxycodone intoxications (▲) and the positive controls (■) in comparison to the model including the whole training set (Figure 1). In addition, a volcanoplot (B) illustrates that acylcarnitines (red circeles) are of high importance for group separation, where a majority has a p(corr) < -0.2 and VIP > 1,5 (R2 = 0.42, Q2 = 0.26).*

**Table S1.** Demographic overview of detected CNS-depressant drugs in the three study groups.

| Analyte[1] | Intoxications (n=376) | Positive Controls (n=364) | Negative Controls (n=207) |
|---|---|---|---|
| Negative drug screening | 0% | 0% | 42% |
| Ethanol | 30% | 16% | 36% |
| Alprazolam | 26% | 10% | 5% |
| Buprenorphine | 4% | 1% | 1% |
| Buspirone | 1% | 0% | 1% |
| Clomethiazole | 0% | 0% | 0% |
| Clonazepam | 3% | 1% | 0% |
| Clozapine | 1% | 0% | 0% |
| Dexmedetomidine | 1% | 1% | 0% |
| Diazepam | 26% | 9% | 7% |
| Fentanyl | 4% | 10% | 0% |
| Haloperidol | 1% | 2% | 1% |
| Hydromorphone | 0% | 0% | 0% |
| Hydroxyzine | 5% | 4% | 2% |
| Ketobemidone | 1% | 0% | 0% |
| Levomepromazine | 3% | 1% | 1% |
| Lithium | 0% | 0% | 0% |
| Lorazepam | 1% | 1% | 0% |
| Methadone | 5% | 1% | 0% |
| Midazolam | 1% | 4% | 0% |
| Morphine | 11% | 9% | 1% |
| Nitrazepam | 1% | 1% | 1% |
| Olanzapine | 3% | 2% | 6% |
| Oxazepam | 13% | 9% | 5% |
| Oxycodone | 100% | 100% | 0% |
| Propiomazine | 7% | 2% | 1% |
| Risperidone | 1% | 1% | 1% |
| Tapentadol | 1% | 0% | 0% |
| Tramadol | 9% | 4% | 1% |
| Zolpidem | 11% | 5% | 1% |
| Zopiclone | 29% | 20% | 7% |

**Table S2.** Cause of Death and Analytical Findings for False positives and False neagiteves (t>5 or t<-5).

| Classified as Control predicted as Intoxication | Primary Cause of Death | Secondary Cause of Death | Positive analytes |
|---|---|---|---|
| Positive Control 1 | Old myocardial infarction | Alcohol dependence syndrome | 0,01 µg/g alprazolam |
| | | | 0,5 µg/g oxycodone |
| | | | 2,5 µg/g venlafaxine |
| | | | 11 µg/g lamotrigine |
| | | | 0,37 µg/g O-desmethylvenlafaxine |
| | | | 0,36 µg/g mirtazapine |
| | | | 0,03 µg/g desmethylmirtazapine |
| | | | 0,08 µg/g duloxetine |
| Positive Control 2 | Hanging | Poisoning by other and unspecified drugs and medicinal substances | 0,24 µg/g alprazolam |

| | | | |
|---|---|---|---|
| | | | 0,34 µg/g Amphetamine |
| | | | 0,25 µg/g diazepam |
| | | | 0,12 µg/g nordazepam |
| | | | 9,3 µg/g ketamine |
| | | | 0,18 µg/g 7-amino-clonazepam |
| | | | 0,54 µg/g MDMA |
| | | | 0,05 µg/g oxazepam |
| | | | 7,1 µg/g oxycodone |
| | | | 0,6 µg/g propranolol |
| | | | 0,04 µg/g temazepam |
| | | | 0,06 µg/g zopiclone |
| | | | 0,03 µg/g zolpidem |
| | | | 0,67 ‰ ethanol i lårblod |
| | | | 0,45 µg/g norfludiazepam |
| | | | 55 ng/g Cyclopropylfentanyl |
| Positive Control 3 | Ill-defined descriptions and complications of heart disease | Drug dependence | 0,39 µg/g alimemazine |
| | | | 0,13 µg/g desmethylalimemazine |
| | | | 0,02 µg/g alprazolam |
| | | | 0,5 µg/g oxycodone |
| | | | 1,2 µg/g venlafaxine |
| | | | 0,16 µg/g O-desmethylvenlafaxine |
| | | | 6,4 µg/g pregabalin |
| | | | 0,13 ‰ ethanol i lårblod |
| Positive Control 4 | Hanging | | 0,83 µg/g citalopram |
| | | | 0,27 µg/g desmethylcitalopram |
| | | | 0,08 µg/g diazepam |
| | | | 0,14 µg/g nordazepam |
| | | | 0,5 µg/g oxycodone |
| | | | 0,05 µg/g propiomazine |
| | | | 0,19 µg/g dihydropropiomazine |
| | | | 0,03 µg/g zopiclone |
| | | | 7,5 µg/g tramadol |
| | | | 1,2 µg/g O-desmethyltramadol |
| | | | 11 µg/g pregabalin |
| | | | 0,20 µg/g amlodipine |
| | | | 0,07 µg/g melatonin |
| Positive Control 5 | Toxic effect of carbon monoxide | | 0,33 µg/g diazepam |
| | | | 0,06 µg/g nordazepam |
| | | | 47 % carboxyhemoglobin |
| | | | 0,24 µg/g oxazepam |

| | | | |
|---|---|---|---|
| | | | 0,03 µg/g oxycodone |
| | | | 4,4 µg/g lamotrigine |
| | | | 0,68 µg/g bupropion |
| | | | 0,54 µg/g duloxetine |
| | | | 0,27 µg/g hydroxybupropion |
| | | | 1,26 ‰ ethanol |

| **Classified as Intoxication, Predicted as Positive Control** | **Primary Cause of Death** | **Secondary Cause of Death** | **Positive analytes** |
|---|---|---|---|
| Inoxication 6 | Toxic effect of alcohol | Diseases of esophagus | 0,006 µg/g oxycodone |
| | | | 3,69 ‰ ethanol |
| Inoxication 7 | Poisoning by other and unspecified drugs and medicinal substances (Multiple organ failure) | | 8,9 µg/g furosemide |
| | | | 0,01 µg/g codeine |
| | | | 0,05 µg/g midazolam |
| | | | 0,08 µg/g oxycodone |
| | | | 54 µg/g acetaminophen |
| Inoxication 8 | Poisoning by other and unspecified drugs and medicinal substances | | 0,07 µg/g 7-amino-clonazepam |
| | | | 0,0023 µg/g clonidine |
| | | | 0,03 µg/g midazolam |
| | | | 0,14 µg/g morphine |
| | | | 0,07 µg/g oxazepam |
| | | | 0,006 µg/g oxycodone |
| | | | 14 µg/g acetaminophen |
| | | | 0,0003 µg/g dexmedetomidine |
| Inoxication 9 | Poisoning by other and unspecified drugs and medicinal substances | | 0,48 µg/g amphetamine |
| | | | 0,80 µg/g citalopram |
| | | | 0,05 µg/g desmethylcitalopram |
| | | | 0,3 µg/g oxycodone |
| | | | 67 µg/g acetaminophen |
| | | | 0,55 µg/g cetirizine |
| | | | 2,7 µg/g pregabalin |
| Inoxication 10 | Poisoning by other and unspecified drugs and medicinal substances | Chronic obstructive pulmonary disease | 0,36 µg/g alimemazine |
| | | | 0,07 µg/g desmethylalimemazine |
| | | | 0,82 µg/g citalopram |

| | | | |
|---|---|---|---|
| | | | 0,06 µg/g desmethylcitalopram |
| | | | 0,009 µg/g clonazepam |
| | | | 0,23 µg/g 7-amino-clonazepam |
| | | | 0,12 µg/g oxycodone |
| | | | 17 µg/g acetaminophen |
| | | | 0,06 µg/g dihydropropiomazine |
| | | | 0,03 µg/g zolpidem |
| | | | 14 µg/g gabapentin |
| Inoxication 11 | Poisoning by other and unspecified drugs and medicinal substances | Other forms of chronic ischemic heart disease | 0,15 µg/g alimemazine |
| | | | 0,13 µg/g desmethylalimemazine |
| | | | 0,02 µg/g oxycodone |
| | | | 0,53 µg/g zopiclone |
| Inoxication 12 | Poisoning by other and unspecified drugs and medicinal substances | | 0,28 µg/g citalopram |
| | | | 0,15 µg/g desmethylcitalopram |
| | | | 0,07 µg/g diazepam |
| | | | 0,07 µg/g nordazepam |
| | | | 11 ng/g fentanyl |
| | | | 0,01 µg/g oxycodone |
| | | | 0,15 µg/g dihydropropiomazine |
| | | | 1,7 µg/g trimetoprim |
| | | | 0,19 µg/g quetiapine |
| | | | 0,05 µg/g zolpidem |
| | | | 0,21 µg/g mirtazapine |
| | | | 0,09 µg/g desmethylmirtazapine |
| | | | 25 µg/g gabapentin |

**Table S3.** Demographic overview of training set and validations set for study cohort.

| | Oxycodone intoxications | | Positive Controls | | Negative Controls | |
|---|---|---|---|---|---|---|
| | Training | Validation | Training | Validation | Training | Validation |
| n | 249 | 126 | 244 | 120 | 132 | 63 |
| Females/males | 116/133 | 44/82 | 77/167 | 46/74 | 29/103 | 15/48 |
| Age (yrs) | 48 (36-62) | 46 (34-57) | 64 (52-72) | 67 (57-77) | 49 (30-60) | 45 (29-56) |
| Body weight (kg) | 85 (71-99) | 87 (73-104) | 76 (62-92) | 74 (62-91) | 73 (65-83) | 74 (62-91) |
| Body Height (cm) | 173 (165-181) | 174 (166-182) | 173 (165-180) | 172 (166-179) | 175 (170-180) | 172 (166-179) |
| Body Mass Index (kg/m$^2$) | 29 (25-33) | 29 (25-34) | 26 (22-30) | 25 (22-29) | 24 (22-27) | 25 (22-29) |
